# Hofbauer Cells in Pregnancies Complicated by Gestational Diabetes Mellitus and Pathological Fetal Growth

**DOI:** 10.1111/aji.70216

**Published:** 2026-02-15

**Authors:** Georgia Fakonti, Georgia Mappa, Nicolas Orsi, Eleanor M. Scott, Beth Holder, Karen Forbes

**Affiliations:** ^1^ Leeds Institute of Cardiovascular and Metabolic Medicine University of Leeds Leeds UK; ^2^ Leeds Institute of Medical Research At St James's University of Leeds Leeds UK; ^3^ Institute of Reproductive Biology Imperial College London London UK

**Keywords:** gestational diabetes mellitus, GDM, Hofbauer cells, LGA, macrophages, placenta

## Abstract

**Problem:**

Gestational diabetes mellitus (GDM) increases the risk of large‐for‐gestational‐age (LGA) birth and long‐term cardiometabolic complications in offspring, particularly in males. These outcomes are associated with altered placental vascularisation, but the underlying mechanisms remain poorly defined. Hofbauer cells (HBCs) are fetal‐origin macrophages located in the villous stroma with established roles in immune regulation and vascularisation.

**Method of Study:**

This study investigated whether HBC abundance and phenotype are associated with fetal growth, fetal sex, and placental vascularisation in term placentae from non‐GDM and GDM pregnancies. Pan‐macrophage (CD14, CD68), HBC‐enriched (FOLR2, VSIG4), M1 (CD86), and M2 (CD163, MRC1) markers were assessed by RT‐qPCR and quantitative immunohistochemistry.

**Results:**

In both non‐GDM and GDM placentae, all markers, except CD86 were detected, supporting an M2‐like HBC phenotype. In GDM placentae, the number of pan‐macrophage (CD68), HBC‐enriched (FOLR2), and M2‐associated (CD163, MRC1) cells were reduced in terminal villi compared with non‐GDM controls (*p* < 0.05; *n* = 13 non‐GDM; *n* = 12 GDM), indicating reduced HBC abundance without phenotypic switching. Reduced expression of HBC‐enriched (FOLR2, VSIG4) and M2‐associated (CD163) transcripts supported these findings (*p* < 0.05; *n* = 18 non‐GDM; *n* = 19 GDM). No further differences were observed following stratification by fetal growth or sex. HBC‐related gene expression correlated positively with the endothelial marker PECAM1/CD31, in both non‐GDM and GDM placentae (*r* ≥ 0.5, *p* < 0.05).

**Conclusions:**

HBCs abundance is reduced in GDM placentae independently of fetal growth or sex, whilst HBC phenotype is preserved. Reduced HBC abundance may contribute to placental vascular alterations that are characteristic of GDM.

## 1 Introduction

Diabetes in pregnancy is considered a high‐risk condition for both mother and offspring. Pregnant women with diabetes, either pre‐existing or arising during pregnancy, known as gestational diabetes mellitus (GDM), have an increased risk of pregnancy complications. These include complications related to birth outcome (stillbirth, miscarriage, preterm birth) and offspring health (later development of obesity, diabetes, and cardiovascular diseases) [1, 2]. Diabetes in pregnancy is also associated with impaired fetal cardiac function and structure, suggesting that cardiovascular diseases may be initiated at early stages of fetal development [3, 4]. Babies born to mothers with diabetes have high risk of pathological fetal growth and are often large for gestational age (LGA; >90th centile), rather than appropriate for gestational age (AGA) [5, 6]. This can lead to several early‐life and long‐term complications, such as obesity and metabolic conditions, with male offspring at particularly high risk [7, 8]. Despite these well‐established clinical associations, the placental immune mechanisms linking maternal diabetes to abnormal fetal growth and adverse pregnancy outcomes remain poorly defined.

Several theories on the origin of fetal growth complications in diabetes have been proposed, including the impact of maternal hyperglycaemia and fetal hyperinsulinemia [9], with even subtle glucose changes during pregnancy reported to impact growth [10, 11]. However, the placental molecular and cellular mechanisms that drive the increased risk of pregnancy complications and LGA offspring remain largely unknown. Research increasingly supports the Developmental Origins of Health and Disease (DOHaD) hypothesis, suggesting that adult disease risk may be programmed during early development [12]. Adverse in utero conditions can shape fetal development through long‐lasting programming effects, including on immune development and function [13]. This positions pregnancy as a critical window during which placental dysfunction may influence both short‐and long‐term outcomes.

Situated at the interface between the maternal and fetal circulations, the placenta is highly sensitive to changes in the maternal environment. Within the placental villous tree, terminal villi are the primary site of maternal‐fetal exchange [14], due to their minimal diffusion distance between maternal and fetal blood. This close proximity may render terminal villi particularly vulnerable to metabolic and inflammatory alterations associated with GDM [15, 16]. Indeed, GDM placentae exhibit alterations in placental inflammatory mediators, villous maturity, placental vasculature (such as reduced branching and increased capillary density), and extracellular matrix [17, 18, 19]. However, the mechanisms driving these changes and their relation to fetal growth in GDM remain elusive.

Hofbauer cells (HBCs)‐ fetal‐origin macrophages, are abundant within terminal villi and are well placed to mediate alterations associated with pathological fetal growth. HBCs play established roles in immune regulation [20], tissue growth, and remodelling [21, 22], and interact closely with mesenchymal and endothelial cells to influence villous development [23]. Given their involvement in inflammation and remodelling, alterations in HBC abundance or phenotype could plausibly contribute to placental dysfunction and abnormal fetal growth in GDM. GDM is characterised by a chronic low‐grade inflammatory state, which may exacerbate placental metabolic and vascular dysfunction [24, 25, 26]. However, HBC abundance and phenotype in terminal villi and their potential role in pathological fetal growth in GDM remain poorly defined.

Macrophages are often categorised into classically‐activated (M1; pro‐inflammatory) and alternatively‐activated (M2; anti‐inflammatory/tissue‐remodelling) phenotypes. Their polarisation from M0 depends on environmental signals that induce expression of different markers (e.g. M1:CD80, CD86 and M2: MRC1, CD163) and secretion of distinct cytokines. Although, this classification is oversimplified and there are many subtypes beyond the M1/M2 dichotomy, HBCs seem to maintain an anti‐inflammatory/tissue remodelling phenotype even in the presence of infection and are considered to maintain a M2 phenotype in diabetes [20, 27]. In this study we quantified the abundance of HBCs in terminal villi and assessed the expression of pan‐macrophage (CD14, CD68), HBC‐enriched (FOLR2 and VSIG4) [28], M1 (CD86), and M2 (CD163 and MRC1) markers in placentae from non‐GDM and GDM pregnancies with AGA or LGA infants. We further examined whether HBC abundance or phenotype was associated with fetal sex or markers of villous vascularisation.

## 2 Materials and Methods

### 2.1 Patient Groups

All research was conducted following ethical approval from North West Regional Ethics Committee (08/H1010/55+5) and London Riverside Research Ethics Committee under the REC reference number 18/LO/0067 and IRAS project ID 234385. Pregnant women were recruited following written informed consent at Leeds Teaching Hospitals Trust or Manchester University NHS Foundation Trust. Placentae from term (≥37 weeks of gestation, live births) singleton uncomplicated pregnancies and pregnancies complicated by GDM with and without pathological fetal growth were studied. GDM was diagnosed by routine oral glucose tolerance test and fetal growth was calculated by estimating the fetal percentile using the World Health Organization growth calculator [29, 30]. Babies born ≥ 90th centile were classified as LGA, and all >10th and <90th centile were classified as AGA. The human tissue processing, data curation and analysis were conducted in accordance with Declaration of Helsinki guidelines, General Data Protection Regulation, and Human Tissue Act. Two independent patient cohorts were utilised for RT‐qPCR and immunohistochemistry studies.

### 2.2 Tissue Processing

Placental tissue was collected within 30–60 min of delivery. Fetal membranes and cord were removed, and the trimmed placenta was weighed. Three full thickness portions, approximately 2 cm^3^, were taken at random from the centre, edge and middle regions of the placenta and washed in phosphate buffered saline (PBS). For immunohistochemistry experiments, a full‐thickness sample (∼1cm^3^) was dissected, washed in PBS, fixed in 10% neutral buffered formalin (HT501128, Sigma‐Aldrich), and embedded in paraffin. For RNA analyses, the chorionic and basal plate were removed before each portion was dissected further into ∼0.25cm^3^ pieces which were refrigerated in RNAlater overnight, snap frozen, and transferred to −80°C for future RNA extraction.

### 2.3 Immunohistochemistry

Tissue sections (5 µm) were mounted on poly‐l‐lysine coated slides and then heated to 60°C for 20 min, followed by incubation in HistoClear (NAT1330D2, SLS) for deparaffinization, and rehydration by passing through graded ethanol baths. Heat‐induced antigen retrieval was performed using sodium citrate (0.01 M, pH 6.0, 2 × 5 min) followed by quenching endogenous peroxidase activity by incubating for 15 min with 3% hydrogen peroxide. Slides were washed with Tris Buffered Saline (TBS) (0.02 M Trisma Base, 0.15 M NaCl, pH = 7.4, 2 × 5 min) and blocked with bovine serum albumin (5% in TBS) for 1 h. Sections were incubated overnight at 4°C with primary antibody or IgG control (Table 1), then washed and incubated with biotinylated secondary antibody for 1 h at room temperature (Table 1). After further TBS washes, avidin peroxidase was applied (A3151, Sigma–Aldrich) (0.02 µM in high salt TBS; 0.005 M Trisma Base, 0.3 M NaCl) for 30 min. ImmPACT DB EqV substrate kit, peroxidase (SK‐4103, Vector lab) used for detection with haematoxylin (1092530500, Merck) counterstaining. Slides were dehydrated, cleared in HistoClear and mounted in DPX (Thermo Fisher Scientific). To confirm that the absence of staining (CD86) in placental tissue was due to antigen absence and not procedure, positive controls (human myometrium) were used.

**TABLE 1:** Antibodies used in immunohistochemistry experiments.

Primary antibodies			
Target	Host species	Final concentration	Manufacturer (catalogue number) (*clone number)* *(RRID number)*
CD163	Rabbit	0.96 µg/ml	Abcam (ab182422) *(EPR19518)* *(RRID: AB_2753196)*
CD68	Rabbit	0.79 µg/ml	Abcam (ab213363) *(EPR20545)* (RRID:AB_2801637)
CD86	Mouse	2 µg/ml	Abcam (ab220188) *(C86/1146)* *(‐)*
MRC1	Mouse	0.2 µg/ml	Proteintech (60143‐1‐Ig) *(2A6A10)* (RRID:AB_2144924)

Secondary antibodies and IgG control			
Antibody	Host species	Dilution	Manufacturer (catalogue number) (*clone number)* *(RRID number*
Biotinylated anti mouse IgG	Goat	5 µg/ml	AAT Bioquest (16729) *(‐)* *(‐)*
Biotinylated anti rabbit IgG	Swine	2.55 µg/ml	Dako (E0353) *(‐)* (RRID:AB_2737292)
Rabbit IgG control	Rabbit	Relative to primary antibody	Vector Laboratories (I‐1000) *(‐)* (RRID:AB_2336355)

Microphotographs obtained using Zeiss Axioscan Z1 slide scanner (20x magnification). Analysis was performed semi‐automated and blinded to any clinical data in QuPath [31]. Colour deconvolution was applied to obtain single stained‐haematoxylin only images to minimise the risk of selection bias. Terminal villi (17/image) were selected based on consistent morphological criteria, such as the size as they are typically smaller than intermediate and stem villi with numerous capillaries and minimal dilation distance from the syncytiotrophoblast layer, with similar total area between groups (Figure S1–S3). A border was manually drawn around each villi, cells within the villus stroma but outside of the fetal vessels were counted, and data were normalised to villous area (mm^2^) using standardised QuPath pipelines [31].

### 2.4 RNA Extraction, cDNA Synthesis, and Quantitative Reverse Transcription PCR (RT‐qPCR)

Total RNA was extracted from 0.25 g of placenta tissue using the mirVana miRNA isolation kit (AM1561, Thermo Fisher Scientific) according to the manufacturer's guidelines for frozen tissue and purified with the RNA clean and concentrator‐5 kit (R1013, Zymo Research), including DNase I treatment before RNA clean‐up according to manufacturer's instructions. The concentration and quality of the eluted RNA was measured with a NanoDrop ND‐1000 Spectrophotometer. RNA (100 ng) was converted to cDNA using the AffinityScript cDNA Synthesis Kit (200436, Agilent) following the manufacturer's instructions. Controls with no reverse transcriptase and no template were also prepared. RT‐qPCR was performed using qPCR Brilliant III SYBR MM with ROX kit (600882, Agilent) with gene specific primers (Integrated DNA Technologies) (Table 2) following the manufacturer's instructions using LightCycler96 (Roche) with the following parameters: 1 cycle (3 min/95°C), 40 cycles (20 s/95°C, 20 s/primer‐specific annealing temperature). A melting curve was included by incubating the samples at 95°C for 1 min, followed by incubation at 55°C for 30 s. The temperature was then increased to 95°C, ramping at 0.2°C/cycle. For each plate, an interplate calibrator sample was included. All samples were run in duplicates (Ct difference between duplicates <0.5) and the ΔΔC_t_ method was used to analyse the data.

**TABLE 2:** List of primers for target and housekeeping genes.

Target gene	Direction	Sequence (5’→3’)	Annealing temperature (°C)
CD68	Forward	CGAGCATCATTCTTTCACCAGCT	60
	Reverse	ATGAGAGGCAGCAAGATGGACC	
CD14	Forward	CTGGAACAGGTGCCTAAAGGAC	60
	Reverse	GTCCAGTGTCAGGTTATCCACC	
CD163	Forward	CGGTCTCTGTGATTTGTAACCAG	55
	Reverse	TACTATGCTTTCCCCATCCATC	
MRC1	Forward	GACGTGGCTGTGGATAAATAAC	55
	Reverse	CAGAAGACGCATGTAAAGCTAC	
FOLR2	Forward	CCTGTACCGAAGACAGAGGC	60
	Reverse	GAGCTGAACCTCCGTTGCT	
VSIG4	Forward	AGAGAGTGTAACAGGACCTT	55
	Reverse	GTCACGTAGAAAGATGGTGA	
PECAM1 (CD31)	Forward	GCTGAGTCTCACAAAGATCTAGGA	57
	Reverse	ATCTGCTTTCCACGGCATCA	

Housekeeping gene	Direction	Sequence (5’→3’)	Annealing temperature (°C)
YWHAZ	Forward	ACTTTTGGTACATTGTGGCTTCAA	55
	Reverse	CCGCCAGGACAAACCAGTAT	
GUSB	Forward	GTCTGCGGCATTTTGTCGG	55
	Reverse	CACACGATGGCATAGGAATGG	
RPLP0	Forward	AGCCCAGAACACTGGTCTC	55
	Reverse	ACTCAGGATTTCAATGGTGCC	

### 2.5 Statistical Analysis

Data distribution was assessed using the Shapiro‐Wilk test. Continuous variables with normal distribution are shown as mean ± standard deviation (SD) and analysed by unpaired t‐test (two‐tailed) or one‐way ANOVA followed by Tukey's post‐hoc test (equal SD) or Brown–Forsythe ANOVA followed by Dunnett T3 post‐hoc test (non‐equal SD). Continuous variables that were not normally distributed are presented as median (q1, q3) and were assessed using Mann‐Whitney (two‐tailed) or Kruskal–Wallis with Dunn's post‐hoc test. Categorical variables are presented as absolute and relative frequencies (%) and analysed by Fisher‐exact or Chi‐squared tests. The interaction between diabetes and fetal growth, or fetal sex was assessed by two‐way ANOVA. When data was not normally distributed, data were transformed using natural logarithm (ln). Data was considered statistically significant when *p* < 0.05. Analysis was performed using GraphPad (v.10.3.1) and correlation matrix was created using the corrplot package in R (v.4.3.1).

## 3 Results

### 3.1 Expression Levels and Phenotype of HBCs in GDM Placentae

**FIGURE 1:**
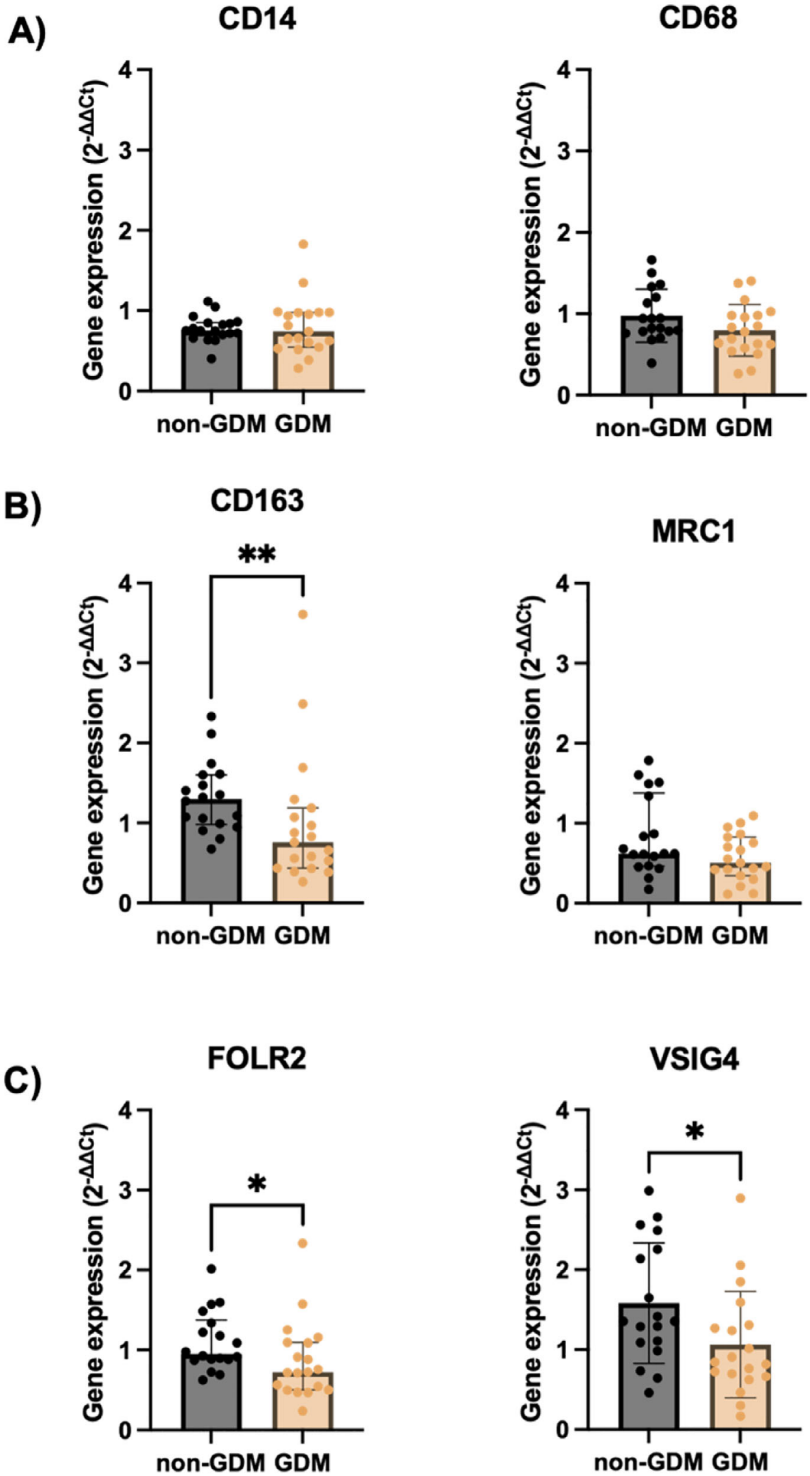
Gene expression of HBC‐ and macrophage‐ enriched markers in non‐GDM and GDM human placental tissue. Gene expression of the (A) pan‐macrophage‐ (CD14 and CD68), (B) M2‐ (CD163 and MRC1), and (C) HBC‐enriched (FOLR2 and VSIG4) markers. Data presented as median with interquartile range and analysed by Mann‐Whitney test (two‐tailed) (CD14; *p*‐value = 0.77, CD163; *p*‐value = 0.01, MRC1; *p*‐value = 0.12, FOLR2; *p*‐value = 0.03) or mean with SD and analysed by unpaired t‐test (two‐tailed) (CD68; *p*‐value = 0.10, VSIG4; *p*‐value = 0.03). Non‐GDM (*n* = 18) and GDM (*n* = 19). *p*‐value <0.05*, ≤0.01**. Expression was normalised to the average of YWHAZ, RPLP0, and GUSB.

**FIGURE 2:**
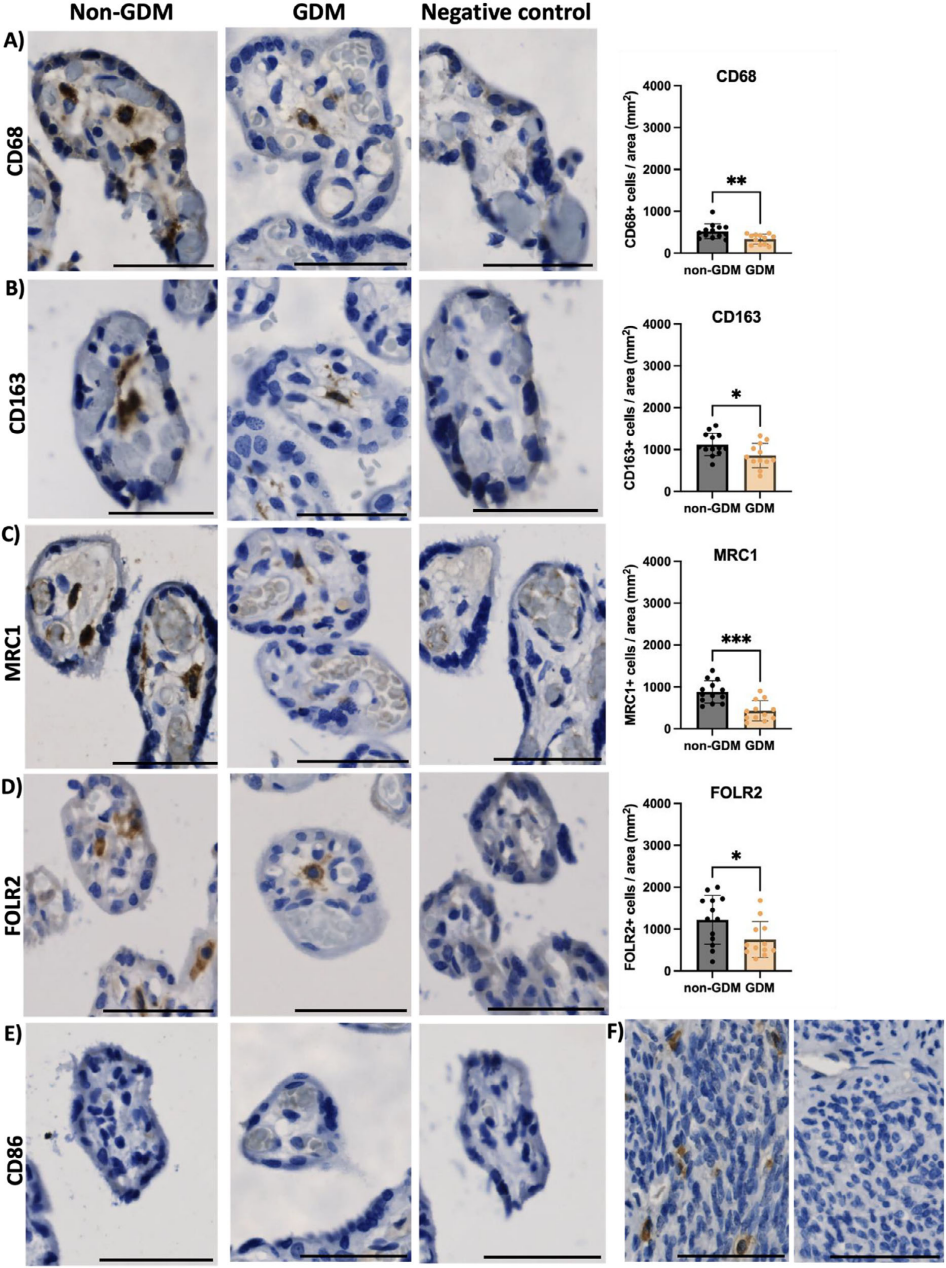
Immunohistochemistry analysis of placental tissue from non‐GDM and GDM pregnancies. HBCs positive for (A) the pan‐macrophage (CD68), (B‐C) M2 (CD163, MRC1), and (D) HBC‐enriched (FOLR2) markers were identified in different locations within the chorionic villi of the placenta in both non‐GDM and GDM pregnancies. Negative controls (secondary antibody only or IgG controls were used). (E) HBCs were negative for the M1 marker CD86. (F) Human uterine samples were used as a positive control (CD86 staining on the left and negative control on the right). Scale bar: 50 µm. Non‐GDM (*n* = 13) and GDM (*n* = 12). Statistical significance at the 0.05 level, *p*‐value <0.05*, *p*‐value ≤0.01**, *p*‐value ≤0.001***.

GDM and non‐GDM participants were comparable with respect to maternal age, parity, smoking status, booking BMI, fetal sex and gestational age at delivery for both RT‐qPCR and immunohistochemistry analyses (Table 3). Pan macrophage (M0; CD68), M2‐associated (MRC1, CD163) and HBC‐enriched (FOLR2) markers were detected at both the transcript (Figure 1A–C) and protein (Figure 2A–D) levels in placental villous tissue from both non‐GDM and GDM pregnancies. The M1‐associated marker CD86, was undetectable in human placental tissue in either group (Figure 2E) despite robust expression in human uterine tissue used as a positive control (Figure 2F).

**TABLE 3:** Demographics of placental samples with/without GDM included in the RT‐qPCR and immunohistochemistry experiments.

RT‐qPCR				Immunohistochemistry			
	Non‐GDM (*n* = 18)	GDM (*n* = 19)	*p*‐value		Non‐GDM (*n* = 13)	GDM (*n* = 12)	*p*‐value
**Maternal age (years)** ^e^	29.8 ± 5.26	32.5 ± 4.05	0.09	**Maternal age (years)** ^e^	31.3 ± 5.59	29.8 ± 6.81	0.56
**Booking BMI (kg/m^2^)** ^e^	29.1 ± 8.66	30.4 ± 5.86^a^	0.61	**Booking BMI (kg/m** ^2^)^g^	28.2 (27.3 30.2)	33.8 (29.4, 35.5)^c^	0.06
**Ethnicity** ^f^			0.12	**Ethnicity** ^f^			0.32
White	13 (72.2)	9 (47.4)		White	11 (84.6)	6 (50.0)	
Black	1 (5.6)	0 (0.0)		Black	0 (0.0)	1 (8.3)	
Asian	4 (22.2)	7 (36.8)		Asian	1 (7.7)	3 (25.0)	
Other	0 (0.0)	3 (15.8)		Other	1 (7.7)	2 (16.7)	
**Smoking status** ^f^			0.23	**Smoking status** ^f^			0.21
QDP	1 (5.6)	0 (0.0)		Ex‐smoker	4 (30.8)	0 (0.0)	
Non‐smoker	16 (88.9)	19 (100.0)		Non‐smoker	7 (53.8)	9 (75.0)	
Smoker	1 (5.6)	0 (0.0)		Smoker	1 (7.7)	1 (8.3)	
				Unknown	1 (7.7)	2 (16.7)	
**Gestational age (days)** ^e^	274.2 ± 6.93	270.9 ± 6.32^b^	0.15	**Gestational age (days)** ^g^	275 (273, 277)	274 (272, 277)	0.51
**Parity** ^f^			0.55	**Parity** ^f^			0.25
0	5 (27.8)	4 (21.1)		0	2 (15.4)	5 (41.7)	
1	6 (33.3)	7 (36.8)		1	5 (38.4)	6 (50.0)	
2	5 (27.8)	3 (15.8)		2	4 (30.8)	1 (8.3)	
3	2 (11.1)	2 (10.5)		3	1 (7.7)	0 (0.0)	
≥4	0 (0.0)	3 (15.8)		≥4	1 (7.7)	0 (0.0)	
**Mode of delivery** ^f^			0.12	**Mode of delivery** ^f^			0.12
SVD	6 (33.3)	4 (21.1)		SVD	0 (0.0)	2 (16.7)	
VD‐ind	0 (0.0)	2 (10.5)		CS‐el	13 (100.0)	10 (83.3)	
CS‐el	11 (61.1)	8 (42.1)					
CS‐em	0 (0.0)	4 (21.2)					
Unknown	1 (5.6)	1 (5.2)					
**Placental weight (g)** ^e^	622.2 ± 140.5^b^	664.6 ± 250	0.54	**Placental weight (g)** ^e^	562 ± 131^d^	758 ± 239	**0.02**
**Birthweight (g)** ^e^	3816 ± 479.8	3725 ± 612.6	0.62	**Birthweight (g)** ^e^	3715 ± 566	3840 ± 409	0.53
**Fetal: placental weight ratio** ^g^	6.1 (4.7, 6.9)^b^	5.6 (4.9, 6.8)	0.16	**Fetal: placental weight ratio** ^g^	6.9 (6.0, 7.5)^d^	4.6 (4.3, 6.5)	**0.01**
**Fetal sex** ^f^			>0.99	**Fetal sex** ^f^			0.43
Male	10 (55.6)	9 (47.4)		Male	9 (69.2)	6 (50.0)	
Female	8 (44.4)	9 (47.4)		Female	4 (30.8)	6 (50.0)	
Unknown	0 (0.0)	1 (5.2)					

Abbreviations: CS‐el, elective caesarean section; CS‐em, emergency caesarean section; QDP, quit during pregnancy; SVD, spontaneous vaginal delivery; VD‐ind, induced vaginal delivery.

^a^ *n* = 18.

^b^ *n* = 17.

^c^ *n* = 10.

^d^ *n* = 15

^e^ mean ± standard deviation.

^f^ frequency (%).

^g^ median (q1, q2).

Statistical significance at the 0.05 level.

At the transcript level, no differences were detected in the pan‐macrophage markers CD14 (*p* = 0.77) or CD68 (*p* = 0.10) expression between groups (Figure 1A). In contrast, immonohistochemistry revealed a significant reduction in the number of CD68 +ve cells, per villous area (mm^2^) in GDM placentae compared with non‐GDM (non‐GDM: 522 ± 178, GDM: 337 ± 119; *p* < 0.01) (Figure 2A).

Expression of the HBC‐enriched markers FOLR2 and VSIG4 was reduced at the mRNA levels in GDM placentae (both *p* = 0.03; Figure 1C), accompanied by fewer HBCs (FOLR2+ cells) per villous area (non‐GDM: 1223 ± 583 cells/mm^2^; GDM: 750 ± 427 cells/mm^2;^ *p* = 0.03; Figure 2D). Similarly, CD163 mRNA expression was lower in GDM placentae (*p* = 0.01; Figure 1B), with a corresponding reduction in the number of CD163+ cells (non‐GDM: 1122 ± 268 cells/mm^2^; GDM: 857 ± 291 cells/mm^2^; *p* = 0.03; Figure 2B). Although MRC1 transcript levels was not different between groups (Figure 1B), the number of MRC1+ cells was significantly reduced in GDM placentae (non‐GDM: 881 ± 267 cells/mm^2^; GDM: 430 ± 242 cells/mm^2^; *p <* 0.001; Figure 2C), potentially suggesting alterations in post transcriptional control of MRC1.

### 3.2 Effect of GDM and Pathological Fetal Growth on HBC Number and Phenotype

**FIGURE 3:**
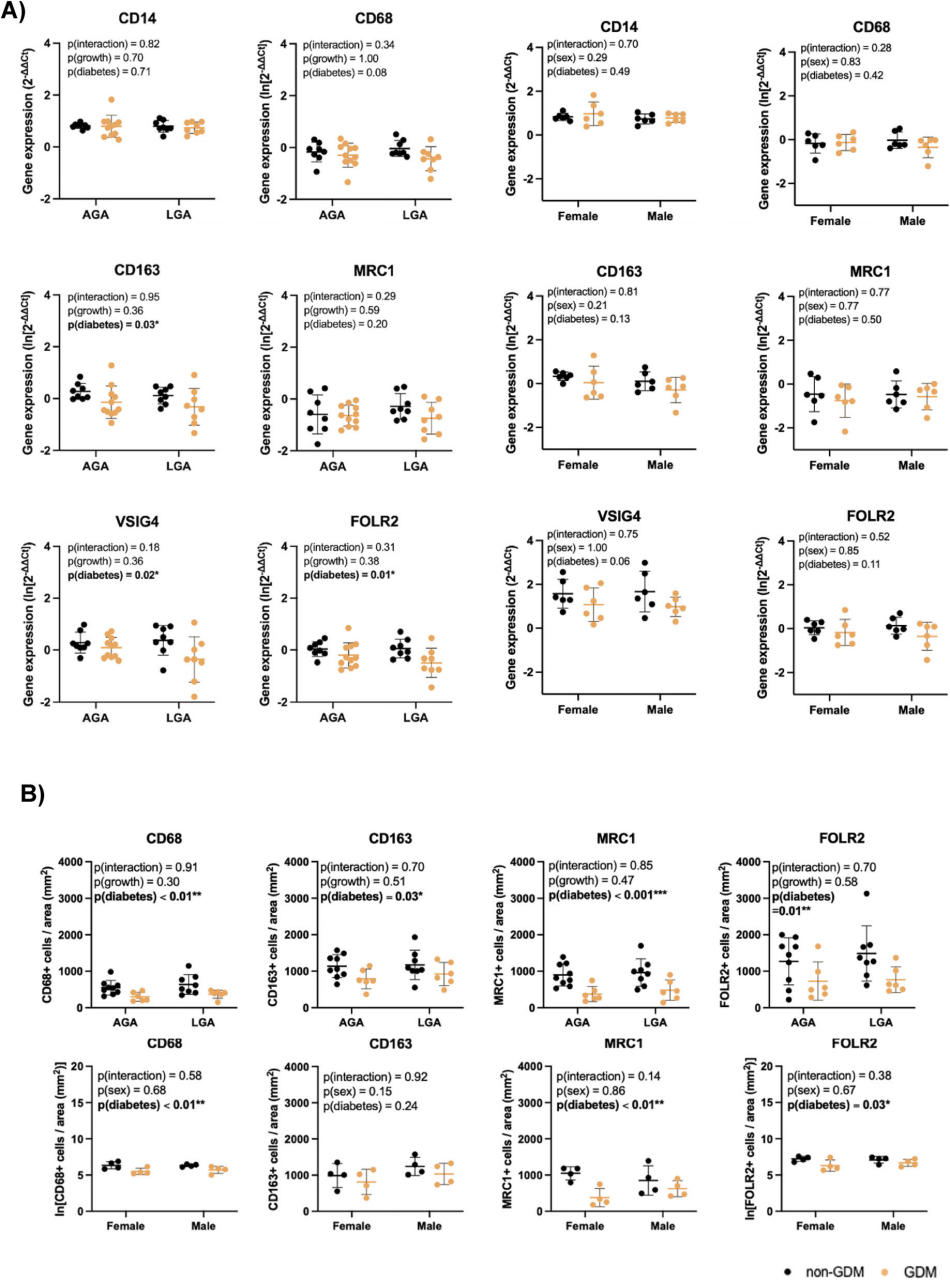
mRNA expression of HBC‐ and macrophage‐ enriched markers and immunohistochemistry analysis of placental tissue classified by pathological fetal growth or sex and diabetes status. (A) Data presented as mean with SD and analysed by two‐way ANOVA, with results displayed in tables. Expression is normalised to YWHAZ, GUSB, and RPLP0 expression. Non‐GDM/AGA (*n* = 8), non‐GDM/LGA (*n* = 8), GDM/AGA (*n* = 11), and GDM/LGA (*n* = 8), non‐GDM/female (*n* = 6), non‐GDM/male (*n* = 6), GDM/female (*n* = 6), and GDM/male (*n* = 6). (B) Analysis of positive cells per surface area between non‐GDM and GDM samples classified by fetal growth and by fetal sex. Data presented as mean with SD and analysed using two‐way ANOVA. Non‐GDM/AGA (*n* = 9), non‐GDM/LGA (*n* = 8), GDM/AGA (*n* = 6), and GDM/LGA (*n* = 6), non‐GDM/female (*n* = 4), non‐GDM/male (*n* = 4), GDM/female (*n* = 4), and GDM/male (*n* = 4). AGA: appropriate for gestational age, LGA: large for gestational age. Bold indicates statistical significance at the 0.05 level, *p*‐value <0.05*, *p*‐value ≤0.01**, *p*‐value ≤0.001***.

To determine whether changes in HBC abundance or phenotype were associated with pathological fetal growth, samples were stratified based by birthweight category (AGA compared to LGA Table S1‐S2). No differences in pan‐macrophage, HBC‐enriched, M1 or M2‐associated markers were observed between AGA and LGA groups in either non‐GDM or GDM placentae (Figure 3).

### 3.3 Fetal Sex Differences in HBC Number and Phenotype

Given reported sex differences in perinatal outcomes [32, 33] and HBC immune responses [34], data were stratified by fetal‐sex (Tables S3 and S4). No sex‐specific differences were detected in transcript or abundance of pan‐macrophage‐ or HBC‐enriched markers in non‐GDM or GDM placentae (Figure 3A &B), indicating that the observed alterations associated with GDM were not sex‐dependent.

### 3.4 Correlation of HBCs and Placental Vascular Markers

**FIGURE 4:**
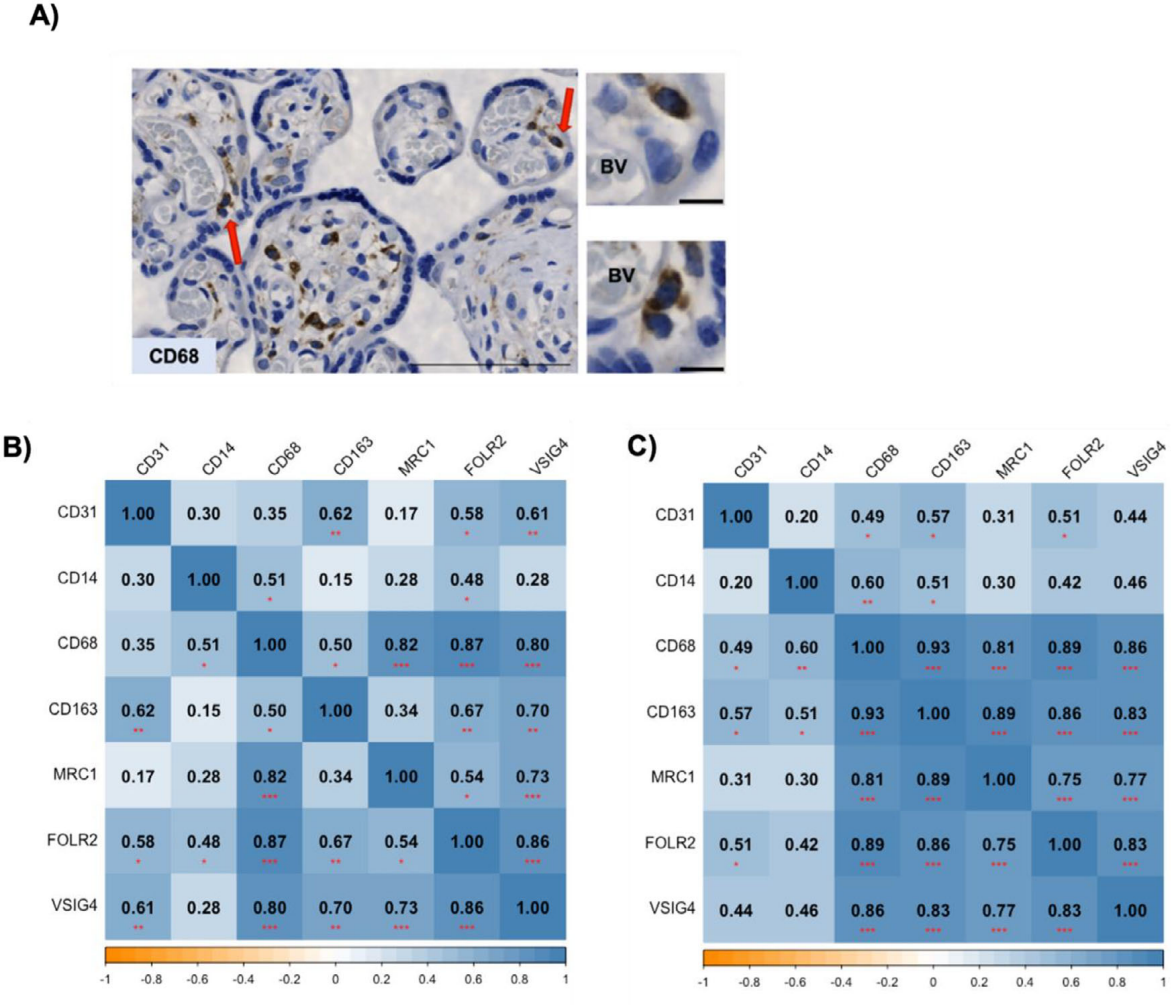
HBCs in placental vascular development. (A) HBCs are found in different locations within the chorionic villi of the placenta in both normal and GDM placentae. Representative image from GDM placenta using the pan‐macrophage marker CD68 (same observations were also made in non‐GDM placentae and with staining for FOLR2, MRC1, and CD163). Red arrows indicate areas presented in detail (right) Scale bar: 100 µm and details scale bar: 10 µm. BV: blood vessel. (B‐C) Correlation matrix of the gene expression of pan‐macrophage (CD14, CD68), M2 (CD163, MRC1), HBC‐enriched markers with the expression of vascular marker, CD31 (also known as PECAM1). Numeric value in each square represents the Pearson's correlation coefficient (colour and numeric value between ‐1; orange to 1; blue) for (B) non‐GDM samples (*n* = 17) and (C) GDM samples (*n* = 18). Significant correlation is represented with red asterisks within each square *p*‐value <0.05*, ≤0.01**, ≤0.001***.

HBCs exhibit an M2 phenotype, and display close proximity to placental vasculature (Figure 4A). As PECAM1/CD31 expression was previously shown to be reduced in GDM compared to non‐GDM placentae [35], we assessed associations between PECAM1 mRNA expression and HBC‐related markers (Figure 4B–C). In non‐GDM placentae, PECAM1 expression correlated positively with CD163 (*r* = 0.62; *p* ≤ 0.01), VSIG4 (*r* = 0.61; *p* ≤ 0.01) and FOLR2 (*r* = 0.58; *p* < 0.05) (Figure 4B). Similar associations were observed in GDM placentae for PECAM1 with FOLR2; (*r* = 0.51; *p* < 0.05) and CD163 (*r* = 0.57; *p* < 0.05) (Figure 4C). Correlation matrices further revealed strong positive relationships between macrophage‐ and HBC‐enriched markers in both non‐GDM (*r* ≥ 5, *r* ≤ 0.86, *p* < 0.05) and GDM placentae (*r* ≥ 0.75, *r* ≤ 0.89; *p* ≤ 0.001) (Figure 4B‐C).

## 4 Discussion

This study investigated HBC numbers and phenotype in term placentae from non‐GDM and GDM pregnancies, exploring their associations with LGA, fetal sex, and placental vascularisation. We demonstrate that HBC, abundance but not phenotype, is reduced in term placentae from pregnancies complicated by GDM. The consistency of our findings at both the transcript and protein level across two independent cohorts strengthens confidence that GDM alters fetal macrophage presence within the villous tree. Importantly, these changes were independent of fetal growth status or fetal sex, indicating that reduced HBC abundance is a fundamental placental consequence of GDM rather than an adaptive response to fetal overgrowth, and is unlikely to contribute to LGA development.

The observed decrease in the number of HBCs in terminal villi of GDM placentae aligns with a previous studies showing a reduction in FOLR2 gene expression in GDM placental lysates [36], although it contrasts with other studies showing increased gene expression of macrophage genes and a higher number of HBCs in placentae from GDM placentae [37, 38, 39, 40]. These discrepancies could arise from variations in demographic characteristics among participants [41, 42], however, it is more likely that they arise from methodological differences. Earlier studies quantified HBCs across whole sections without controlling for villous subtype or villous area or villous type‐factors that can strongly influence cell density. We focused on terminal villi as they are more likely to be highly influenced by components of the maternal circulation. By restricting our analysis to morphologically matched terminal villi and normalising to villous area, we minimised sampling bias and obtained a more accurate and anatomically meaningful measure of HBC abundance. This methodological clarity likely explains the more coherent patterns observed across our datasets. The overall reduction in HBC‐enriched gene expression and HBC number suggests a disruption in fetal macrophage presence that could influence villous immune homeostasis.

Consistent with previous literature, our study found no evidence of M1 polarisation. M1 markers were undetectable in the placenta villous tree, whereas M2 macrophage markers were consistently detected at both mRNA and protein level, supporting the concept that HBCs exhibit an M2 anti‐inflammatory, tissue‐remodelling phenotype even in the context of metabolic stress [27, 43]. The preserved M2‐like phenotype may reflect intrinsic fetal programming to protect the developing fetus from an excessive inflammatory environment that is potentially mediated by epigenetic regulation of M1 and M2 genes [44]. It is likely that a reduction in HBCs is associated with functional impacts in GDM placentae that are associated with M2 phenotype macrophages. Although we investigated HBCs in third trimester, their roles in early pregnancy may be distinct [45] and potentially pivotal in influencing the placental vasculature and fetal growth [28]. While GDM is typically diagnosed at 24–28 weeks, studying pregnancies affected by pregestational diabetes could provide insight into how early metabolic disturbances influence HBCs.

The placenta is a highly vascular organ and changes in placental vascular development in GDM placentae have been reported [46]. HBCs are anatomically positioned to influence villous angiogenesis, and their M2‐like phenotype is compatible with pro‐angiogenic and tissue remodelling roles. We observed strong positive correlations between HBC‐enriched markers and the endothelial marker PECAM1/CD31 in both groups, consistent with earlier reports that HBCs can release angiogenic factors which assist endothelial network formation [47]. However, these relationships are correlative. Term placentae represent the end of gestation and do not permit inference regarding earlier developmental periods when angiogenic pathways are most active. Whilst one study in first trimester placentae, also reported a positive correlation between the number of vascular structures and HBCs [22], this correlation does not show causation, and further functional studies are needed to investigate this.

Macrophages are essential for preventing infection and HBCs have been shown to respond to inflammatory stimuli [34]. If HBCs act in this manner, the observed reduction in HBC numbers in GDM placentae could potentially contribute to an increased risk of fetal infection. Hyperglycaemia is known to impair innate immune responses outside pregnancy, by enhancing macrophage pro‐inflammatory cytokine secretion and diminishing their phagocytic and pathogen‐fighting abilities [48]. Notably, a reduction in HBC numbers is also observed in conditions like chorioamnionitis [49] and severe preeclampsia [50], which are associated with a higher risk of infection and neurodevelopmental *sequelae* in neonates [51, 52]. Consistent with this notion, infants of GDM mothers are more likely to experience infectious morbidity [53]. Therefore, preserving both the quantity and anti‐inflammatory properties of HBCs in GDM is likely vital for protecting the fetus and ensuring long‐term offspring health. Although there has been some investigation into the role of HBCs in infection in normal pregnancies [20], studies specifically examining HBC responses to infection in GDM are lacking.

Despite these interesting findings, this study has some limitations. Because tissue was obtained from archived collections, the numbers of available samples, particularly for immunohistochemistry, remained modest. Moreover, glucose control data were not available, preventing assessment of the influence of glycaemic severity on HBCs. Finally, our work focused on HBC gene expression and abundance, rather than functional roles, and mechanistic implications cannot be inferred from these data. Functional studies across gestation are required to determine the biological significance of reduced HBC abundance.

In summary, our findings indicate that GDM is associated with reduced HBC abundance in terminal villi while preserving their M2‐like phenotype. These alterations may contribute to the broader alterations in placental villous homeostasis observed in GDM. Further mechanistic studies are needed to determine the developmental timing and functional impact of these changes.

## Supporting information




**Supplementary Figure 1**: Comparable villous surface areas between participants in the non‐GDM and GDM groups. (A–L) The selected surface area of 17 villi per sample was comparable across samples within the non‐GDM and GDM group and average area for each participant was comparable across the two groups. (A&B) For CD68 analysis, data are presented as median with interquartile range and analysed by Kruskal–Wallis with Dunn's post hoc test ((A) non‐GDM, *p* = 0.85; (B) GDM, p = 0.98) and (C) for average area comparison, data presented as mean with SD and analysed by unpaired t‐test (two‐tailed) (CD68; *p* = 0.21). (D&E) For CD163 analysis, data are presented as median with interquartile range and analysed by Kruskal–Wallis with Dunn's post hoc test ((D) non‐GDM, *p* = 0.94; (E) GDM, *p* = 0.98) and (F) for average area comparison, data are presented as mean with SD and analysed by unpaired t‐test (two‐tailed) (CD163; *p* = 0.85). (G&H) For MRC1 analysis, data are presented as median with interquartile range and analysed by Kruskal–Wallis with Dunn's post hoc test ((G) non‐GDM, *p* = 0.50; (H) GDM, *p* = 0.59) and (I) for average area comparison, data are presented as mean with SD and analysed by unpaired t‐test (two‐tailed) (MRC1; *p* = 0.52). (J&K) For FOLR2 analysis of non‐GDM samples (J), data are presented as median with interquartile range and analysed by Kruskal–Wallis with Dunn's post hoc test (*p* = 0.79) and for GDM samples (K) as mean with SD and analysed by one‐way ANOVA with Tukey post hoc test (*p* = 0.70) and (L) for average area comparison, data are presented as mean with SD and analysed by unpaired t‐test (two‐tailed) (*p* = 0.66). (A,B,D,E,G,H,J,K) Each bar represents a different sample and dots represent surface area of villi (µm^2^). Non‐GDM (*n* = 13) and GDM (*n* = 12). **Supplementary Figure 2**: Comparable villous surface areas between participants in the non‐GDM and GDM groups with AGA and LGA offspring, and among the four study groups. (A–T) The selected surface area of 17 villi per sample was comparable across samples within the same group and between the average values for each participant across the four groups. (A–D) For CD68 analysis, data are presented as median with interquartile range and analysed by Kruskal–Wallis with Dunn's post hoc test ((A) non‐GDM/AGA, *p* = 0.82; (B) non‐GDM/LGA, *p* = 0.23; (D) GDM/LGA, *p* = 0.98) or mean with SD and analysed by one‐way ANOVA with Tukey post hoc test ((C) (GDM/AGA; *p* = 0.95). (E) Analysis of the average surface area of 17 villi per sample between groups was performed using Kruskal–Wallis with Dunn's post hoc test and data are presented as median with interquartile range (*p* = 0.12). (F–I) For CD163 analysis, data are presented as median with interquartile range and analysed by Kruskal–Wallis with Dunn's post hoc test ((F) non‐GDM/AGA, *p* = 0.78; (G) GDM/AGA, *p* = 0.94) or mean with SD and analysed by one‐way ANOVA with Tukey post hoc test ((H) non‐GDM/LGA, *p* = 0.93; (I) GDM/LGA, *p* = 0.71). (J) Analysis of the average surface area of 17 villi per sample between groups was performed using one‐way ANOVA with Tukey post hoc test and data are presented as mean with SD (*p* = 0.97). (K–N) For MRC1 analysis, data are presented as median with interquartile range and analysed by Kruskal–Wallis with Dunn's post hoc test ((K) non‐GDM/AGA, *p* = 0.58; (L) non‐GDM/LGA, *p* = 0.63; (M) GDM/AGA, *p* = 0.55) or mean with SD and analysed by one‐way ANOVA with Tukey post hoc test ((N) GDM/LGA, *p* = 0.84). (O) Analysis of the average surface area of 17 villi per sample between groups was performed using one‐way ANOVA with Tukey post hoc test and data are presented as mean with SD (*p* = 0.17). (P–S) For FOLR2 analysis, data are presented as median with interquartile range and analysed by Kruskal–Wallis with Dunn's post hoc test ((P) non‐GDM/AGA, *p* = 0.75; (Q) non‐GDM/LGA, *p* = 0.74), mean with SD and analysed by one‐way ANOVA with Tukey post hoc test ((R) GDM/AGA, *p* = 0.55), or mean with SD and analysed by Brown–ForsytheANOVA test with Dunnett T3 post hoc test ((S) GDM/LGA, *p* = 0.10). (T) Analysis of the average surface area of 17 villi per sample between groups was performed using Kruskal–Wallis with Dunn's post hoc test and data are presented as median with interquartile range (*p* = 0.33). (A‐D, F‐I, K‐N, P‐S) Each bar represents a different sample and dots represent surface area of villi (µm^2^). Non‐GDM/AGA (*n* = 9), non‐GDM/LGA (*n* = 8), GDM/AGA (*n* = 6), and GDM/LGA (*n* = 6). **Supplementary Figure 3:** Comparable villous surface areas between participants in the non‐GDM and GDM groups with female and male offspring, and among the four study groups. (A‐T) The selected surface area of 17 villi per sample was comparable across samples within the same group and between the average values for each participant across the four groups. (A–D) For CD68 analysis, data are presented as median with interquartile range and analysed by Kruskal–Wallis with Dunn's post hoc test ((B) non‐GDM/Male, *p* = 0.21; (D) GDM/Male, *p* = 0.75) or mean with SD and analysed by one‐way ANOVA with Tukey post hoc test (A) non‐GDM/Female; *p* = 0.08, (C) GDM/Female; *p* = 0.85). (E) Analysis of the average surface area of 17 villi per sample between groups was performed using one‐way ANOVA with Tukey post hoc test and data are presented as mean with SD (*p* = 0.31). (F–I) For CD163 analysis, data are presented as median with interquartile range and analysed by Kruskal–Wallis with Dunn's post hoc test ((F) non‐GDM/Female; *p* = 0.86, (H) GDM/Female; *p* = 0.84) or mean with SD and analysed by one‐way ANOVA with Tukey post hoc test ((G) non‐GDM/Male; *p* = 0.66, (I) GDM/Male; *p* = 0.69). (J) Analysis of the average surface area of 17 villi per sample between groups was performed using one‐way ANOVA with Tukey post hoc test and data are presented as mean with SD (*p* = 0.77). (K–N) For MRC1 analysis, data are presented as median with interquartile range and analysed by Kruskal–Wallis with Dunn's post hoc test ((K) non‐GDM/Female, *p* = 0.45; (M) GDM/Female, *p* = 0.31) or mean with SD and analysed by one‐way ANOVA with Tukey post hoc test ((L) non‐GDM/Male, *p* = 0.64; (N) GDM/Male, *p* = 0.79). (O) Analysis of the average surface area of 17 villi per sample between groups was performed using one‐way ANOVA with Tukey post hoc test and data are presented as mean with SD (*p* = 0.20). (P–S) For FOLR2 analysis, data are presented as median with interquartile range and analysed by Kruskal–Wallis with Dunn's post hoc test ((P) non‐GDM/Female, *p* = 0.39; (Q) non‐GDM/Male, *p* = 0.67), or mean with SD and analysed by one‐way ANOVA with Tukey post hoc test ((R) GDM/Female, *p* = 0.30; (S) GDM/Male, *p* = 0.84). (T) Analysis of the average surface area of 17 villi per sample between groups was performed using Kruskal–Wallis with Dunn's post hoc test and data are presented as median with interquartile range (*p* = 0.86). (A–D; F–I; K–N; P–S) Each bar represents a different sample and dots represent surface area of villi (µm^2^). Non‐GDM/Female (*n* = 4), non‐GDM/Male (*n* = 4), GDM/Female (*n* = 4), and GDM/Male (*n* = 4).

**Supplementary Table 1**: Demographics of placental samples with/without GDM and pathological fetal growth used for the RT‐qPCR experiments. **Supplementary Table 2**: Demographics of placental samples with/without GDM and pathological fetal growth used for the immunohistochemistry experiments. **Supplementary Table 3**: Demographics of a subgroup of placental samples with/without GDM categorised by fetal sex in the RT‐qPCR experiments. **Supplementary Table 4**: Demographics of a subgroup of placental samples with/without GDM categorised by fetal sex in the immunohistochemistry experiments.

## Data Availability

All data underlying the results are available as part of the article and no additional source data are required.
