# Supplementary material for: Hofbauer Cells in Pregnancies Complicated by Gestational Diabetes Mellitus and Pathological Fetal Growth
Source: Am J Reprod Immunol. 2026 Feb 15;95(2):e70216. doi: 10.1111/aji.70216 (PMC12906864; doi:10.1111/aji.70216)
Supplement: Supplementary file 1 — Supplementary Figure 1: Comparable villous surface areas between participants in the non‐GDM and GDM groups. (A–L) The selected surface area of 17 villi per sample was comparable across samples within the non‐GDM and GDM group and average area for each participant was comparable across the two groups. (A&B) For CD68 analysis, data are presented as median with interquartile range and analysed by Kruskal–Wallis with Dunn's post hoc test ((A) non‐GDM, p = 0.85; (B) GDM, p = 0.98) and (C) for average area comparison, data presented as mean with SD and analysed by unpaired t‐test (two‐tailed) (CD68; p = 0.21). (D&E) For CD163 analysis, data are presented as median with interquartile range and analysed by Kruskal–Wallis with Dunn's post hoc test ((D) non‐GDM, p = 0.94; (E) GDM, p = 0.98) and (F) for average area comparison, data are presented as mean with SD and analysed by unpaired t‐test (two‐tailed) (CD163; p = 0.85). (G&H) For MRC1 analysis, data are presented as median with interquartile range and analysed by Kruskal–Wallis with Dunn's post hoc test ((G) non‐GDM, p = 0.50; (H) GDM, p = 0.59) and (I) for average area comparison, data are presented as mean with SD and analysed by unpaired t‐test (two‐tailed) (MRC1; p = 0.52). (J&K) For FOLR2 analysis of non‐GDM samples (J), data are presented as median with interquartile range and analysed by Kruskal–Wallis with Dunn's post hoc test (p = 0.79) and for GDM samples (K) as mean with SD and analysed by one‐way ANOVA with Tukey post hoc test (p = 0.70) and (L) for average area comparison, data are presented as mean with SD and analysed by unpaired t‐test (two‐tailed) (p = 0.66). (A,B,D,E,G,H,J,K) Each bar represents a different sample and dots represent surface area of villi (µm2). Non‐GDM (n = 13) and GDM (n = 12). Supplementary Figure 2: Comparable villous surface areas between participants in the non‐GDM and GDM groups with AGA and LGA offspring, and among the four study groups. (A–T) The selected surface area of 17 vill [file AJI-95-e70216-s002.docx]

**Supplementary Figure 1
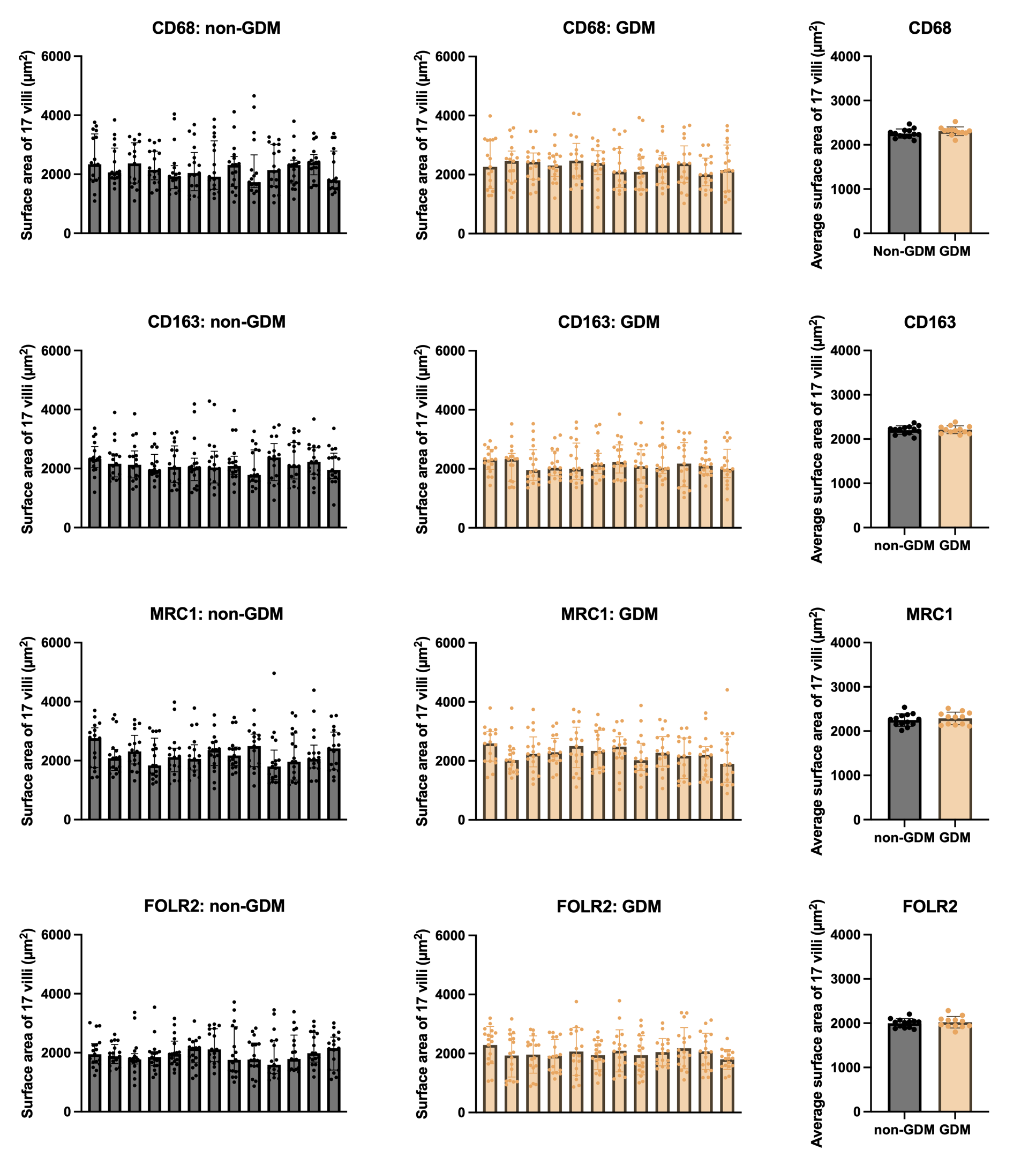
**

**C)**

**F)**

**I)**

**L)**

**K)**

**J)**

**H)**

**G)G)**

**E)**

**D)**

**A)**

**B)**

**Supplementary Figure 1: Comparable villous surface areas between participants in the non-GDM and GDM groups.**

**(A-L**) The selected surface area of 17 villi per sample was comparable across samples within the non-GDM and GDM group and average area for each participant was comparable across the two groups. (**A&B**) For CD68 analysis, data are presented as median with interquartile range and analysed by Kruskal-Wallis with Dunn’s post hoc test (**A**) non-GDM, p=0.85; (**B)** GDM, p=0.98) and (**C**) for average area comparison, data presented as mean with SD and analysed by unpaired t-test (two-tailed) (CD68; p=0.21). (**D&E**) For CD163 analysis, data are presented as median with interquartile range and analysed by Kruskal-Wallis with Dunn’s post hoc test ((**D)** non-GDM, p=0.94; (E) GDM, p=0.98) and (**F**) for average area comparison, data are presented as mean with SD and analysed by unpaired t-test (two-tailed) (CD163; p=0.85). (**G&H**) For MRC1 analysis, data are presented as median with interquartile range and analysed by Kruskal-Wallis with Dunn’s post hoc test ((**G**)non-GDM, p=0.50; **(H)** GDM, p=0.59) and (**I**) for average area comparison, data are presented as mean with SD and analysed by unpaired t-test (two-tailed) (MRC1; p=0.52). (**J&K**) For FOLR2 analysis of non-GDM samples (**J**), data are presented as median with interquartile range and analysed by Kruskal-Wallis with Dunn’s post hoc test (p=0.79) and for GDM samples **(K)** as mean with SD and analysed by one-way ANOVA with Tukey post hoc test (p=0.70) and (**L**) for average area comparison, data are presented as mean with SD and analysed by unpaired t-test (two-tailed) (p=0.66). (**A**,**B,D,E,G,H,J,K**) Each bar represents a different sample and dots represent surface area of villi (μm^2^). Non-GDM (n=13) and GDM (n=12).

**Supplementary Figure 2**

**E)**

**D)**

**C)**

**B)**


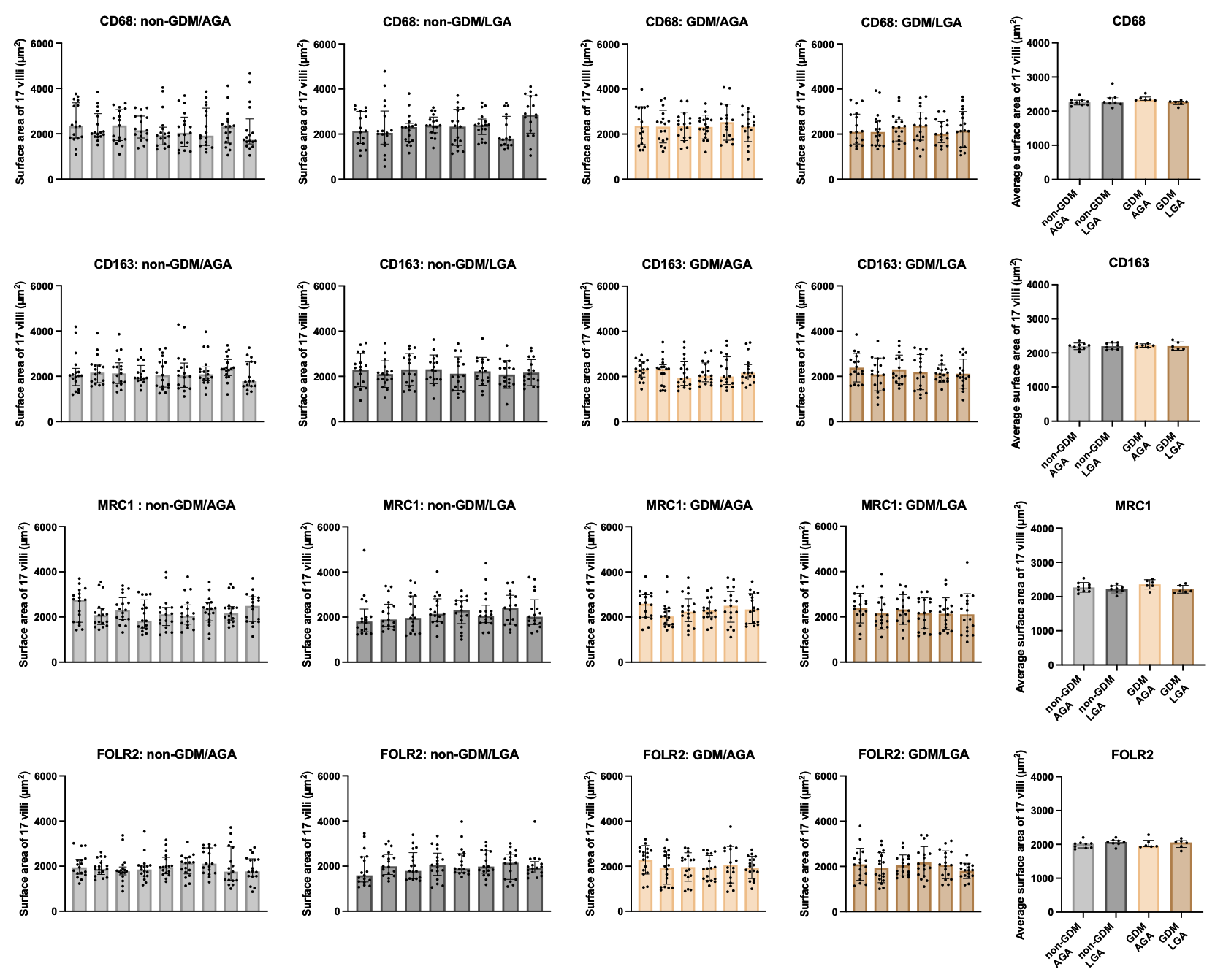


**J)**

**O)**

**T)**

**S)**

**Q)**

**R)**

**P)**

**N)**

**L)**

**M)**

**K)**

**I)**

**G)**

**H)**

**F)**

**A)**

**Supplementary Figure 2: Comparable villous surface areas between participants in the non-GDM and GDM groups with AGA and LGA offspring, and among the four study groups.**

***(A-T)*** *The selected surface area of 17 villi per sample was comparable across samples within the same group and between the average values for each participant across the four groups.* ***(A-D)*** *For CD68 analysis, data are presented as median with interquartile range and analysed by Kruskal-Wallis with Dunn’s post hoc test (****(A)*** *non-GDM/AGA, p=0.82;* ***(B)*** *non-GDM/LGA, p=0.23;* ***(D)*** *GDM/LGA, p=0.98) or mean with SD and analysed by one-way ANOVA with Tukey post hoc test (****(C)****GDM/AGA; p= 0.95).* ***(E)*** *Analysis of the average surface area of 17 villi per sample between groups was performed using Kruskal-Wallis with Dunn’s post hoc test and data are presented as median with interquartile range (p=0.12).* ***(F-I)*** *For CD163 analysis, data are presented as median with interquartile range and analysed by Kruskal-Wallis with Dunn’s post hoc test (****(F)*** *non-GDM/AGA, p=0.78;* ***(G)*** *GDM/AGA, p=0.94) or mean with SD and analysed by one-way ANOVA with Tukey post hoc test (****(H)*** *non-GDM/LGA, p=0.93;* ***(I)****GDM/LGA, p=0.71). (****J****) Analysis of the average surface area of 17 villi per sample between groups was performed using one-way ANOVA with Tukey post hoc test and data are presented as mean with SD (p=0.97). (****K-N****) For MRC1 analysis, data are presented as median with interquartile range and analysed by Kruskal-Wallis with Dunn’s post hoc test (****(K)*** *non-GDM/AGA, p=0.58; (****L****) non-GDM/LGA, p=0.63;* ***(M)*** *GDM/AGA, p=0.55) or mean with SD and analysed by one-way ANOVA with Tukey post hoc test (****(N)*** *GDM/LGA, p=0.84). (****O****) Analysis of the average surface area of 17 villi per sample between groups was performed using one-way ANOVA with Tukey post hoc test and data are presented as mean with SD (p=0.17). (****P-S****) For FOLR2 analysis, data are presented as median with interquartile range and analysed by Kruskal-Wallis with Dunn’s post hoc test (****(P)*** *non-GDM/AGA, p=0.75; (****Q****) non-GDM/LGA, p=0.74), mean with SD and analysed by one-way ANOVA with Tukey post hoc test ((****R****) GDM/AGA, p=0.55), or mean with SD and analysed by Brown-Forsythe ANOVA test with Dunnett T3 post hoc test ((****S****) GDM/LGA, p=0.10). (****T****) Analysis of the average surface area of 17 villi per sample between groups was performed using Kruskal-Wallis with Dunn’s post hoc test and data are presented as median with interquartile range (p=0.33).* ***(A-D, F-I, K-N, P-S)*** *Each bar represents a different sample and dots represent surface area of villi (μm^2^). Non-GDM/AGA (n=9), non-GDM/LGA (n=8), GDM/AGA (n=6), and GDM/LGA (n=6).*

**Supplementary Figure 3**

**K)**

**A)**

**F)**

**O)**

**T)**

**E)**

**J)**

**I)**

**G)**

**H)**

**D)**

**B)**

**C)**


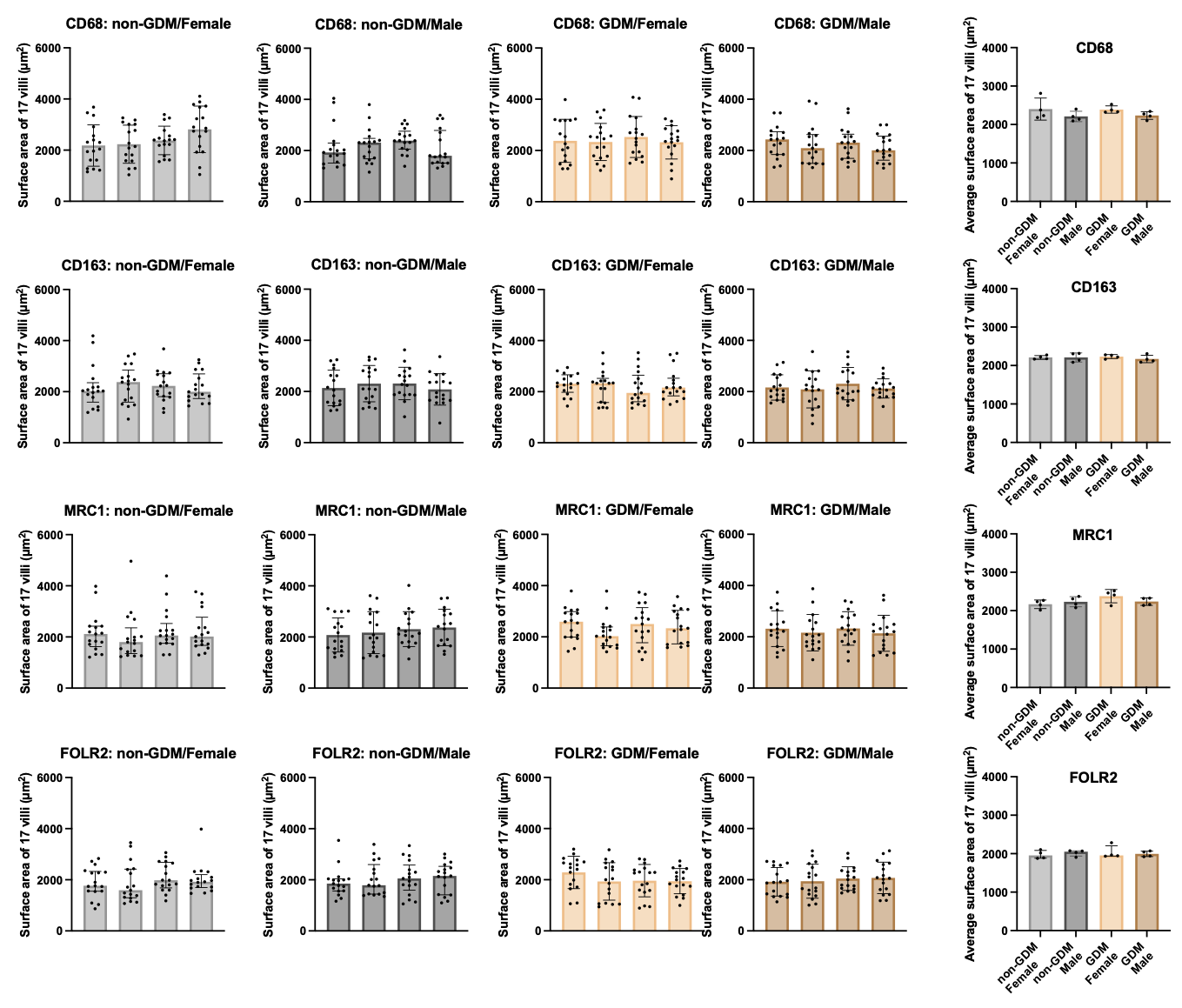


**P)**

**R)**

**Q)**

**S)**

**N)**

**L)**

**M)**

**Supplementary Figure 3 Comparable villous surface areas between participants in the non-GDM and GDM groups with female and male offspring, and among the four study groups.**

*(****A-T****) The selected surface area of 17 villi per sample was comparable across samples within the same group and between the average values for each participant across the four groups. (****A-D****) For CD68 analysis, data are presented as median with interquartile range and analysed by Kruskal-Wallis with Dunn’s post hoc test (****(B)*** *non-GDM/Male, p=0.21;* ***(D)*** *GDM/Male, p=0.75) or mean with SD and analysed by one-way ANOVA with Tukey post hoc test* ***(A****) non-GDM/Female; p=0.08,* ***(C)*** *GDM/Female; p= 0.85). (****E****) Analysis of the average surface area of 17 villi per sample between groups was performed using one-way ANOVA with Tukey post hoc test and data are presented as mean with SD (p=0.31). (****F-I****) For CD163 analysis, data are presented as median with interquartile range and analysed by Kruskal-Wallis with Dunn’s post hoc test ((****F****) non-GDM/Female; p=0.86,* ***(H)*** *GDM/Female; p=0.84) or mean with SD and analysed by one-way ANOVA with Tukey post hoc test* ***((G)*** *non-GDM/Male; p=0.66,* ***((I)****GDM/Male; p= 0.69). (****J****) Analysis of the average surface area of 17 villi per sample between groups was performed using one-way ANOVA with Tukey post hoc test and data are presented as mean with SD (p=0.77). (****K-N****) For MRC1 analysis, data are presented as median with interquartile range and analysed by Kruskal-Wallis with Dunn’s post hoc test (****(K)*** *non-GDM/Female, p=0.45;* ***(M)*** *GDM/Female, p=0.31) or mean with SD and analysed by one-way ANOVA with Tukey post hoc test (****(L)*** *non-GDM/Male, p=0.64; (****N****) GDM/Male, p= 0.79). (****O****) Analysis of the average surface area of 17 villi per sample between groups was performed using one-way ANOVA with Tukey post hoc test and data are presented as mean with SD (p=0.20). (****P-S****) For FOLR2 analysis, data are presented as median with interquartile range and analysed by Kruskal-Wallis with Dunn’s post hoc test ((****P****) non-GDM/Female, p=0.39;* ***(Q)*** *non-GDM/Male, p=0.67), or mean with SD and analysed by one-way ANOVA with Tukey post hoc test (****(R)*** *GDM/Female, p=0.30; (****S)*** *GDM/Male, p=0.84). (****T****) Analysis of the average surface area of 17 villi per sample between groups was performed using Kruskal-Wallis with Dunn’s post hoc test and data are presented as median with interquartile range (p=0.86). (***A**-**D; F-I; K-N; P-S***) Each bar represents a different sample and dots represent surface area of villi (μm^2^). Non-GDM/Female (n=4), non-GDM/Male (n=4), GDM/Female (n=4), and GDM/Male (n=4).*
