# Supplementary material for: Hofbauer Cells in Pregnancies Complicated by Gestational Diabetes Mellitus and Pathological Fetal Growth
Source: Am J Reprod Immunol. 2026 Feb 15;95(2):e70216. doi: 10.1111/aji.70216 (PMC12906864; doi:10.1111/aji.70216)
Supplement: Supplementary file 2 — Supplementary Table 1: Demographics of placental samples with/without GDM and pathological fetal growth used for the RT‐qPCR experiments. Supplementary Table 2: Demographics of placental samples with/without GDM and pathological fetal growth used for the immunohistochemistry experiments. Supplementary Table 3: Demographics of a subgroup of placental samples with/without GDM categorised by fetal sex in the RT‐qPCR experiments. Supplementary Table 4: Demographics of a subgroup of placental samples with/without GDM categorised by fetal sex in the immunohistochemistry experiments. [file AJI-95-e70216-s001.docx]

**Supplementary Table 1: Demographics of placental samples with/without GDM and pathological fetal growth used for the RT-qPCR experiments.**

|  | **Non-GDM**  **AGA  (n=8)** | **Non-GDM**  **LGA  (n=8)** | **GDM**  **AGA  (n=11)** | **GDM**  **LGA  (n=8)** | **p-value** |
| --- | --- | --- | --- | --- | --- |
| **Maternal age (years)^1^** | 28.4±  6.97 | 31.8±  3.73 | 33.9±  4.95 | 34.9±  4.42 | 0.07 |
| **Booking BMI (kg/m^2^)^2^** | 29.3 (22.4,  36.9) | 25.5 (20.8, 40.5) | 29.0 (26.5,  36.6) | 33.4 (30.0,  35.2) ^a^ | 0.78 |
| **Ethnicity^3^**   \| White \| \| --- \| \| Black \| \| Asian \| \| Other \| | \| 7 (87.5) \| \| --- \| \| 0 (0.0) \| \| 1 (12.5) \| \| 0 (0.0) \| | \| 6 (75.0) \| \| --- \| \| 1 (12.5) \| \| 1 (12.5) \| \| 0 (0.0) \| | \| 4 (36.4) \| \| --- \| \| 1 (9.0) \| \| 5 (45.5) \| \| 1 (9.0) \| | \| 5 (62.5) \| \| --- \| \| 0 (0.0) \| \| 2 (25.0) \| \| 1 (12.5) \| | 0.42 |
| **Smoking status^3^**   \| QDP \| \| --- \| \| Non-smoker \| \| Smoker \| | \| 1 (12.5) \| \| --- \| \| 6 (75.0) \| \| 1 (12.5) \| | \| 0 (0.0) \| \| --- \| \| 8 (100.0) \| \| 0 (0.0) \| | \| 0 (0.0) \| \| --- \| \| 11 (100.0) \| \| 0 (0.0) \| | \| 0 (0.0) \| \| --- \| \| 8 (100.0) \| \| 0 (0.0) \| | 0.14 |
| **Gestational age (days)^2^** | 272.5 (271.0,  276.8) | 269.5 (266.5,  274.0) | 268.0 (266.0,  270.0) | 271.5 (267.5,  274.0) ^b^ | 0.09 |
| **Parity^3^**   \| 0 \| \| --- \| \| 1 \| \| 2  3  ≥4 \| | \| 1 (12.5) \| \| --- \| \| 3 (37.5) \| \| 2 (25.0) \| \| 2 (25.0) \| \| 0 (0.0) \| | \| 2 (25.0) \| \| --- \| \| 3 (37.5) \| \| 3 (37.5) \| \| 0 (0.0) \| \| 0 (0.0) \| | \| 1 (9.0) \| \| --- \| \| 4 (36.4) \| \| 3 (27.3) \| \| 3 (27.3) \| \| 0 (0.0) \| | \| 1 (12.5) \| \| --- \| \| 4 (50.0) \| \| 0 (0.0) \| \| 1 (12.5) \| \| 2 (25.0) \| | 0.55 |
| **Mode of delivery^3^**   \| SVD \| \| --- \| \| VD-ind \| \| CS-el \| \| CS-em \| \| Unknown \| | \| 1 (12.5) \| \| --- \| \| 0 (0.0) \| \| 6 (75.0) \| \| 0 (0.0) \| \| 1 (12.5) \| | \| 2 (25.0) \| \| --- \| \| 0 (0.0) \| \| 6 (75.0) \| \| 0 (0.0) \| \| 0 (0.0) \| | \| 4 (36.4) \| \| --- \| \| 2 (18.2) \| \| 4 (36.4) \| \| 1 (9.0) \| \| 0 (0.0) \| | \| 0 (0.0) \| \| --- \| \| 0 (0.0) \| \| 4 (50.0) \| \| 2 (25.0) \| \| 2 (25.0) \| | 0.13 |
| **Placental weight (g)^1^** | 533.3 ±  84.51 ^a^ | 700.0 ±  121.6 | 556.1 ±  134.4 | 838.7 ±  305.9 ^a^ | **0.01**^c^ |
| **Birthweight (g) ^1^** | 3372 ±  175.7 | 4103 ±  359.4 | 3333 ±  302.9 | 4359 ±  349.1 | **<0.001**^d^ |
| **Fetal sex^3^**   \| Male \| \| --- \| \| Female \| \| Unknown \| | \| 5 (62.5) \| \| --- \| \| 3 (37.5) \| \| 0 (0.0) \| | \| 3 (37.5) \| \| --- \| \| 5 (62.5) \| \| 0 (0.0) \| | \| 5 (45.4) \| \| --- \| \| 6 (54.6) \| \| 0 (0.0) \| | \| 3 (37.5) \| \| --- \| \| 3 (37.5) \| \| 2 (25.0) \| | 0.47 |
| **Fetal: placental  weight ratio^2^** | 6.1 (5.6, 7.1)^a^ | 6.1 (5.1, 6.5) | 6.1 (5.4, 7.1) | 5.1 (4.6, 5.3)^a^ | 0.19 |
| **Fetal percentile^2^** | 47.30  (35.30,  58.48) | 94.45  (91.23,  97.50) | 43.90 (38.90,  83.20) | 97.50 (96.08, 97.50) ^b^ | **<0.001** ^e^ |

^1^mean ± standard deviation, ^2^median (q1, q3), ^3^frequency (%), ^a^n=7, ^b^n=6, QDP; quite during pregnancy, SVD; spontaneous vaginal delivery, VD-ind; induced vaginal delivery, CS-el; elective caesarean section, CS-em; emergency caesarean section. Bold indicates statistical significance at the 0.05 level, adjusted p-value <0.05*, ≤0.01 **, ≤0.001***, ^c^(GDM/AGA vs. GDM/LGA^**^), ^d^(non-GDM/AGA vs. non-GDM/LGA^***^, GDM/AGA vs. GDM/LGA^***^) ^e^(non-GDM/AGA vs. non-GDM/LGA^**^, GDM/AGA vs. GDM/LGA^**^).

**Supplementary Table 2: Demographics of placental samples with/without GDM and pathological fetal growth used for the immunohistochemistry experiments.**

|  | **Non-GDM**  **AGA (n=9)** | **Non-GDM**  **LGA (n=8)** | **GDM**  **AGA  (n=6)** | **GDM**  **LGA  (n=6)** | **p-value** |
| --- | --- | --- | --- | --- | --- |
| **Maternal age (years)^1^** | 30.7 ±  5.74 | 33.6 ±  4.72 | 29.0 ±  6.57 | 30.7 ±  7.55 | 0.55 |
| **Booking BMI (kg/m^2^) ^1^** | 30.4 ±  4.35 | 24.1±  2.9 | 32.5 ±  3.4 | 33 ±  4.54 ^a^ | **0.001** **^c^** |
| **Ethnicity^2^**   \| White \| \| --- \| \| Black \| \| Asian \| \| Other \| | \| 8 (88.9) \| \| --- \| \| 0 (0.0) \| \| 1 (11.1) \| \| 0 (0.0) \| | \| 6 (75.0) \| \| --- \| \| 0 (0.0) \| \| 0 (0.0) \| \| 2 (25.0) \| | \| 3 (50.0) \| \| --- \| \| 1 (16.7) \| \| 1 (16.7) \| \| 1 (16.7) \| | \| 3 (50.0) \| \| --- \| \| 0 (0.0) \| \| 2 (33.3) \| \| 1 (16.7) \| | 0.29 |
| **Smoking status^2^**   \| Ex-smoker \| \| --- \| \| Non-smoker \| \| Smoker \| \| Unknown \| | \| 3 (33.3) \| \| --- \| \| 4 (44.4) \| \| 1 (11.1) \| \| 1 (11.1) \| | \| 1 (12.5) \| \| --- \| \| 7 (87.5) \| \| 0 (0.0) \| \| 0 (0.0) \| | \| 0 (0.0) \| \| --- \| \| 5 (83.3) \| \| 0 (0.0) \| \| 1 (16.7) \| | \| 0 (0.0) \| \| --- \| \| 4 (66.7) \| \| 1 (16.7) \| \| 1 (16.7) \| | 0.46 |
| **Gestational age (days)^3^** | 274 (273,  277) | 276 (273,  285.8) | 273.5  (270,  276.5) | 274.5  (270.3,  277) | 0.70 |
| **Parity^2^**   \| 0 \| \| --- \| \| 1 \| \| 2 \| \| 3 \| \| ≥4 \| | \| 1 (11.1) \| \| --- \| \| 2 (22.2) \| \| 4 (44.4) \| \| 1 (11.1) \| \| 1 (11.1) \| | \| 3 (37.5) \| \| --- \| \| 4 (50.0) \| \| 1 (12.5) \| \| 0 (0.0) \| \| 0 (0.0) \| | \| 3 (50.0) \| \| --- \| \| 2 (33.3) \| \| 1 (16.7) \| \| 0 (0.0) \| \| 0 (0.0) \| | \| 2 (33.3) \| \| --- \| \| 4 (66.7) \| \| 0 (0.0) \| \| 0 (0.0) \| \| 0 (0.0) \| | 0.42 |
| **Mode of delivery^2^**   \| NVD \| \| --- \| \| CS-el \| | \| 0 (0.0) \| \| --- \| \| 9 (100.0) \| | \| 0 (0.0) \| \| --- \| \| 8 (100.0) \| | \| 1 (16.7) \| \| --- \| \| 5 (83.3) \| | \| 1 (16.7) \| \| --- \| \| 5 (83.3) \| | 0.32 |
| **Placental weight (g)^1^** | 544 ±  142^d^ | 642 ±  159 | 661 ±  180 | 856 ±  266 | 0.05 |
| **Birthweight (g) ^1^** | 3455 ±  369 | 4238 ±  361 | 3563 ±  340 | 4118 ±  259 | **<0.001^e^** |
| **Fetal sex^3^**   \| Male \| \| --- \| \| Female \| | \| 7 (77.8) \| \| --- \| \| 2 (22.2) \| | \| 4 (50.0) \| \| --- \| \| 4 (50.0) \| | \| 2 (33.3) \| \| --- \| \| 4 (66.7) \| | \| 4 (66.7) \| \| --- \| \| 2 (33.3) \| | 0.34 |
| **Fetal: placental weight ratio^1^** | 6.8 ± 1.7^d^ | 6.9 ± 1.3 | 5.8 ± 2.1 | 5.2 ± 1.6 | 0.21 |
| **Fetal percentile^3^** | 55.2  (21.4,  75.9) | 96.9  (91.1,  97.5) | 62.8  (38.6,  86.2) | 94.7  (91.1,  97.5) | **<0.001^f^** |

^1^mean ± standard deviation, ^2^frequency (%), ^3^median (q1, q2), ^a^n=4, ^b^n=2. ^c^(non-GDM/AGA vs. non-GDM/LGA^**^ & non-GDM/LGA vs GDM/LGA^**^), NVD; normal vaginal delivery, CS-el; elective caesarean section, ^d^n=7, ^e^(non-GDM/AGA vs. non-GDM/LGA^***^ & GDM/AGA vs. GDM/LGA^*^), ^f^(non-GDM/AGA vs. non-GDM/LGA^**^ & GDM/AGA vs. GDM/LGA^*^). Bold indicates statistical significance at the 0.05 level, adjusted p-value <0.05*, ≤0.01 **, ≤0.001***.

**F)**

**H)**

**E)**

**G)**

**H)**

**F)**

**G)**

**E)**

**Supplementary Table 3: Demographics of a subgroup of placental samples with/without GDM categorised by fetal sex in the RT-qPCR experiments.**

|  | **Non-GDM**  **Female  (n=6)** | **Non-GDM**  **Male  (n=6)** | **GDM**  **Female (n=6)** | **GDM**  **Male (n=6)** | **p-value** |
| --- | --- | --- | --- | --- | --- |
| **Maternal age (years)^1^** | 31.50±  6.442 | 28.50±  4.231 | 31.67±  5.465 | 31.33±  2.422 | 0.64 |
| **Booking BMI (kg/m^2^)^2^** | 34.17 (22.8,  37.4) | 24.53 (21.5, 31.6) | 32.80 (27.7,  39.1) | 27.25 (23.0,  31.3) | 0.38 |
| **Ethnicity^3^**   \| White \| \| --- \| \| Black \| \| Asian \| \| Other \| | \| 3 (50.0) \| \| --- \| \| 1 (16.7) \| \| 2 (33.3) \| \| 0 (0.0) \| | \| 4 (66.7) \| \| --- \| \| 0 (0.0) \| \| 2 (33.3) \| \| 0 (0.0) \| | \| 3 (50.0) \| \| --- \| \| 0 (0.0) \| \| 2 (33.3) \| \| 1 (16.7) \| | \| 2 (33.3) \| \| --- \| \| 0 (0.0) \| \| 4 (66.7) \| \| 0 (0.0) \| | 0.76 |
| **Smoking status^3^**  QDP  Non-smoker | \| 1 (16.7) \| \| --- \| \| 5 (83.3) \| | \| 0 (0.0) \| \| --- \| \| 6 (100) \| | \| 0 (0.0) \| \| --- \| \| 6 (100.0) \| | \| 0 (0.0) \| \| --- \| \| 6 (100.0) \| | 0.37 |
| **Gestational age (days)^2^** | 272.5 (269.5,  280.8) | 269.5 (267.5,  278.8) | 269.5 (266.0,  281.0) | 268.5 (266.8,  275.8) | 0.86 |
| **Parity^3^**   \| 0 \| \| --- \| \| 1 \| \| 2  3 \| | \| 1 (16.7) \| \| --- \| \| 1 (16.7) \| \| 2 (33.3) \| \| 2 (33.3) \| | \| 3 (50.0) \| \| --- \| \| 1 (16.7) \| \| 2 (33.3) \| \| 0 (0.0) \| | \| 2 (33.3) \| \| --- \| \| 2 (33.3) \| \| 1 (16.7) \| \| 1 (16.7) \| | \| 1 (16.7) \| \| --- \| \| 3 (50.0) \| \| 1 (16.7) \| \| 1 (16.7) \| | 0.86 |
| **Mode of delivery^3^**   \| SVD \| \| --- \| \| VD-ind \| \| CS-el \| \| CS-em \| | \| 2 (33.3) \| \| --- \| \| 0 (0.0) \| \| 4 (66.7) \| \| 0 (0.0) \| | \| 2 (33.3) \| \| --- \| \| 0 (0.0) \| \| 4 (66.7) \| \| 0 (0.0) \| \|  \| | \| 2 (33.3) \| \| --- \| \| 1 (16.7) \| \| 2 (33.3) \| \| 1 (16.7) \| \|  \| | \| 2 (33.3) \| \| --- \| \| 1 (16.7) \| \| 1 (16.7) \| \| 2 (33.3) \| | 0.60 |
| **Placental weight (g)^1^** | 508.8±  68.91 | 679.2±  168.7 | 607.1±  130.6 | 731.8 ±  231.7 | 0.12 |
| **Birthweight (g) ^1^** | 3425 ±  268.0 | 4073 ±  473.6 | 3454 ±  394.2 | 3879 ±  709.6 | 0.08 |
| **Fetal: placental weight ratio^2^** | 6.6 (6.1, 7.4) | 6.2 (4.9, 7.8) | 5.7 (5.0, 6.5) | 5.2 (4.6, 6.5) | 0.15 |
| **Fetal percentile^2^** | 47.5 (23.0,  70.2) | 93.5 (86.2,  96.2) | 54.3 (39.5,  81.2) | 91.0 (57.0, 94.8) | 0.11 |

^1^mean ± standard deviation, ^2^median (q1, q3), ^3^frequency (%), QDP; quite during pregnancy, SVD; spontaneous vaginal delivery, VD-ind; induced vaginal delivery, CS-el; elective caesarean section, CS-em; emergency caesarean section. Statistical significance at the 0.05 level.

**Supplementary Table 4: Demographics of a subgroup of placental samples with/without GDM categorised by fetal sex in the immunohistochemistry experiments.**

|  | **Non-GDM**  **Female  (n=4)** | **Non-GDM**  **Male  (n=4)** | **GDM**  **Female  (n=4)** | **GDM**  **Male  (n=4)** | **p-value** |
| --- | --- | --- | --- | --- | --- |
| **Maternal age (years)^1^** | 35.3 ± 4.57 | 32.3 ± 3.10 | 26.8 ± 5.62 | 32.0 ± 8.45 | 0.27 |
| **Booking BMI (kg/m^2^)^1^** | 26.1 ± 3.14 | 26.4 ± 3.83 | 31.0 ± 2.96 | 32.4 ± 3.89 | 0.05 |
| **Ethnicity^2^**   \| White \| \| --- \| \| Black \| \| Asian \| \| Other \| | \| 3 (75.0) \| \| --- \| \| 0 (0.0) \| \| 0 (0.0) \| \| 1 (25.0) \| | \| 4 (100.0) \| \| --- \| \| 0 (0.0) \| \| 0 (0.0) \| \| 0 (0.0) \| | \| 2 (50.0) \| \| --- \| \| 1 (25.0) \| \| 0 (0.0) \| \| 1 (25.0) \| | \| 1 (25.0) \| \| --- \| \| 0 (0.0) \| \| 3 (75.0) \| \| 1 (25.0) \| | 0.11 |
| **Smoking status^2^**  Non-smoker  Ex-smoker  Smoker  Unknown | \| 2 (50.0) \| \| --- \| \| 2 (50.0) \| \| 0 (0.0) \| \| 0 (0.0) \| | \| 3 (75.0) \| \| --- \| \| 0 (0.0) \| \| 1 (25.0) \| \| 0 (0.0) \| | \| 3 (75.0) \| \| --- \| \| 0 (0.0) \| \| 0 (0.0) \| \| 1 (25.0) \| | \| 3 (75.0) \| \| --- \| \| 1 (25.0) \| \| 0 (0.0) \| \| 0 (0.0) \| | 0.60 |
| **Gestational age (days)^3^** | 274.5 (273.3, 286.3) | 273.0 (266.3, 285.8) | 274.0 (271.5, 279.5) | 274.0 (268.8, 276.3) | 0.91 |
| **Parity^3^**   \| 0 \| \| --- \| \| 1 \| \| 2  3 \| \| ≥4 \| | \| 2 (50.0) \| \| --- \| \| 1 (25.0) \| \| 1 (25.0) \| \| 0 (0.0) \| \| 0 (0.0) \| | \| 0 (0.0) \| \| --- \| \| 2 (50.0) \| \| 1 (25.0) \| \| 0 (0.0) \| \| 1 (25.0) \| | \| 3 (75.0) \| \| --- \| \| 1 (25.0) \| \| 0 (0.0) \| \| 0 (0.0) \| \| 0 (0.0) \| | \| 0 (0.0) \| \| --- \| \| 3 (75.0) \| \| 1 (25.0) \| \| 0 (0.0) \| \| 0 (0.0) \| | 0.32 |
| **Mode of delivery^2^**   \| NVD \| \| --- \| \| CS-el \| | \| 0 (0.0) \| \| --- \| \| 4 (100.0) \| | \| 0 (0.0) \| \| --- \| \| 4 (100.0) \| | \| 1 (25.0) \| \| --- \| \| 3 (75.0) \| | \| 0 (0.0) \| \| --- \| \| 4 (100.0) \| | >0.99 |
| **Placental weight (g)^1^** | 531 ±  151 | 589 ±  112 | 620 ±  212 | 758 ±  211 | 0.35 |
| **Birthweight (g) ^1^** | 4119 ±  510 | 3838 ±  697 | 3725 ±  278 | 3795 ±  455 | 0.71 |
| **Fetal: placental weight  ratio^3^** | 7.7 (6.7, 9.9) | 6.35 (6.0, 7.2) | 6.1 (4.8, 8.9) | 4.6 (4.3, 7.0) | 0.15 |
| **Fetal percentile^3^** | 94.5 (77.0,  97.5) | 90.6 (38.3,  95.9) | 84.3 (52.6,  87.9) | 91.1 (50.2, 91.6) | 0.33 |

^1^mean ± standard deviation, ^2^frequency (%), ^3^median (q1, q3), SVD; spontaneous vaginal delivery, CS-el; elective caesarean section. Statistical significance at the 0.05 level.
